# Modeling the QSAR of ACE-Inhibitory Peptides with ANN and Its Applied Illustration

**DOI:** 10.1155/2012/620609

**Published:** 2011-06-09

**Authors:** Ronghai He, Haile Ma, Weirui Zhao, Wenjuan Qu, Jiewen Zhao, Lin Luo, Wenxue Zhu

**Affiliations:** ^1^School of Food and Biological Engineering, Jiangsu University, 301 Xuefu Road, Zhenjiang, Jiangsu 212013, China; ^2^School of Food and Biological Engineering, Henan University of Science and Technology, 48 Xiyuan Road, Luoyang, Henan 471003, China

## Abstract

A quantitative structure-activity relationship (QSAR) model of angiotensin-converting enzyme- (ACE-) inhibitory peptides was built with an artificial neural network (ANN) approach based on structural or activity data of 58 dipeptides (including peptide activity, hydrophilic amino acids content, three-dimensional shape, size, and electrical parameters), the overall correlation coefficient of the predicted versus actual data points is *R* = 0.928, and the model was applied in ACE-inhibitory peptides preparation from defatted wheat germ protein (DWGP). According to the QSAR model, the C-terminal of the peptide was found to have principal importance on ACE-inhibitory activity, that is, if the C-terminal is hydrophobic amino acid, the peptide's ACE-inhibitory activity will be high, and proteins which contain abundant hydrophobic amino acids are suitable to produce ACE-inhibitory peptides. According to the model, DWGP is a good protein material to produce ACE-inhibitory peptides because it contains 42.84% of hydrophobic amino acids, and structural information analysis from the QSAR model showed that proteases of Alcalase and Neutrase were suitable candidates for ACE-inhibitory peptides preparation from DWGP. Considering higher DH and similar ACE-inhibitory activity of hydrolysate compared with Neutrase, Alcalase was finally selected through experimental study.

## 1. Introduction

In recent years, some progress have been made in bioinformatics study of functional peptide preparation, such as comparing active peptide sequences in database, hydrolysis enzyme choosing, simulated hydrolysis, activity prediction of hydrolysate, and so forth [1–6]. However, these studies were all based on a known sequence of protein. In fact, bioinformatics application on peptide is still difficult because the majority of proteins have complicated components or unknown sequences.

Besides comparing characterized peptide sequences in databases, peptide quantitative structure-activity relationship (QSAR) models could also be used in peptide bioinformatics study. QSAR models are mathematical functions that describe the relationship between activity and chemical structure expressed by variables. Such models are applied both to predict activity of untested chemical structures and to predict the chemical structure of compounds with specific activity [7]. Several QSAR models have been investigated on ACE-inhibitory peptides. These models were built based on different amino acid descriptors or multivariate statistical regression techniques, such as multiple linear regressions (MLR) or partial least square regression (PLSR), and 3D-QSAR was also used to describe ACE-inhibitory peptide [8–18]. Recently, quantitative sequence-activity model (QSAM) was employed in ACE-inhibitory peptide study [19]. In addition, docking and virtual screening of ACE-inhibitory dipeptides technique was studied, but it also needs experimental verification [20]. 

An artificial neural network (ANN) is an interdisciplinary technique, involving biology, mathematics, physics, electronics, and computer technology. It is a kind of information processing system based on imitation of the structure and function of brain networks. It is the theoretical model of the human neural network. ANN technique can simulate any nonlinear process; therefore, it can avoid the linear deficiencies [15, 16, 18]. 

In this study, illustrated by preparation of ACE-inhibitory peptides from defatted wheat germ protein, a QSAR model was built with ANN. The structural characteristics of the ACE-inhibitory peptides were investigated according to the model. Based on the structural characteristics analysis and experimental result of DWGP digestion, appropriate protease was selected to produce high-activity ACE-inhibitory peptides from DWGP isolates.

## 2. Materials and Methods

### 2.1. Materials and Chemicals

Defatted wheat germ protein was purchased from Man Tian Xue Flour Industry (Henan, China). Alcalase 2.4 LFG (2.670 AU/g) and medium temperature amylase 480 L (527.50 KNU/g) were purchased from Novo Co. (Shanghai, China). Angiotensin I-converting enzyme (ACE; EC 3.4.15.1) was purchased from Sigma Chemical Co. (St. Louis, MO, USA). N-(3[2-Furyl]Acryloyl)-Phe-Gly-Gly (FAPGG) was purchased from Fluka Chemical Corp. (Milwaukee, WI, U.S.A.). All the other reagents were in analytical purity grade.

### 2.2. Instruments

The instruments used were as follows: thermostat-controlled water-bath (model HH), Jintan Zhongda Instruments Co., Ltd. (Jintan, Jiangsu, China); pH meter (model PHS-3C), Shanghai Precision & Scientific instrument Co., Ltd. (Shanghai, China); electrothermal blast drying oven, Shanghai Laboratory Instrument Works Co., Ltd. (Shanghai, China); Agilent 1100 HPLC, Agilent Technologies Inc. (Santa Clara, CA, U.S.A.); SPX-250B biochemistry incubator, Changzhou Guohua Electric Co., Ltd. (Changzhou, China); Multiskan Spectrum Microplate Reader, Thermo Scientific Inc. (Hudson, NH, U.S.A.).

### 2.3. Methods

#### 2.3.1. DWGP Isolates Preparation

DWGP isolates were prepared according to the method described by XIN Zhi-hong [21] with minor modifications. DWGP was dispersed in 0.2 mol/L NaCl solution at the ratio of 1 : 10 (w/v) and stirred for 30 min at ambient temperature. Then, the suspension's pH was adjusted to 9.5 by using 1 mol/L NaOH. After stirring for 30 min, the suspension was centrifuged at 8000 r/m for 20 min at 4°C. The supernatant was adjusted to pH 7.0 with 1.0 mol/L HCl, then 0.3% (v/v) *α*-amylase was added in. After stirring for 180 min at 70°C, it was adjusted to pH 4.0 with 1.0 mol/L HCl to precipitate the protein, and the solution was centrifuged at 8000 r/m for 20 min. The precipitate was washed several times with distilled water (pH 4.0), and was then dispersed in a small amount of distilled water, then it was adjusted to pH 7.0 with 0.1 mol/L NaOH. The dispersed precipitate was dried by spraying dryer (model B290, BUCHI Laboratory Equipment Ltd., Switzerland) to get DWGP isolates.

#### 2.3.2. Hydrolysis of DWGP in a Batch Reactor

Ten grams of DWGP was dispersed in 1 L distilled water and was digested in batch by Alcalase at pH 9.0, 50°C or by Neutrase at pH 7.0 at 50°C, both at the enzyme/substrate mass ratio of 8% ([E]/[S]). Samples were collected at 0.5, 1, 1.5, 2, 3, 4, and 5 h and were immediately heated in a boiling water bath for 10 min. After cooling, the samples were centrifuged at 10,000 r/m for 15 min, and the supernatants were diluted with distilled water to determine their ACE-inhibitory activities.

#### 2.3.3. Building of QSAR Model on ACE-Inhibitory Peptides

In this study, *Z* descriptor was used to predict the ACE-inhibitory activity of peptides, amino acids descriptor selected *Z*-scales, *Z*_1_, *Z*_2_, and *Z*_3_ means the hydrophilic amino acids, three-dimensional shape, size, and electrical parameters, respectively (Table 1) [22]. Three-layer back propagation (BP) neural network was used to establish a QSAR model to describe relationships between peptide structure and activity.

Fifty-eight kinds of ACE-inhibitory peptides (dipeptides) samples and their activity data (50% inhibitory concentration on ACE, i.e., IC_50_ value) were used in the text and were shown in Table 2. Each dipeptide corresponds to a dependent variable (log[1/IC_50_]) and six independent variables (*Z* parameters).

Because of the quite different physical meaning of the input parameters, the following formula was used in this study to make the sample sets data normalized so as to accelerate network convergence and overfitting:

(1) $$Z^{\prime }=\frac{Z-Z_{\min\;}}{Z_{\max\;}-Z_{\min\;}},$$

where, *Z*′ is the normalized value of the operator, *Z* value is the *Z* operator, *Z*_max_ and *Z*_min_ are the maximum and minimum of the *Z* operator vector before being normalized for each sample.

**Figure 1:**
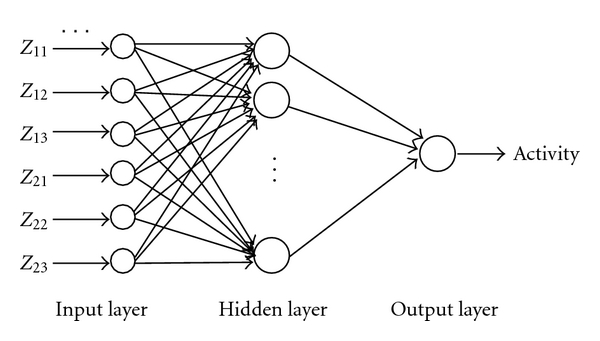
Diagram of BP-ANN model of ACE-inhibitory peptides' QSAR.

39 dipeptides were randomly selected as study samples in the neural network model, the rest were test samples. Each of two peptides corresponding to 6 *Z* operators as a BP neural network input vector. The network output vector is the activity value.  Figure 1 is the structure of BP network model.

A three-level BP neural network model was built using MATLAB neural network tool (from Matrix Laboratory). Transfer functions of neurons in hidden layer and output layer were Tansig function and Purelin function, respectively. Because the BP neural network is not easily converged or easily falls into local minimum, the following steps were applied to avoid it: (1) network training algorithm using gradient descent momentum Traingdm, (2) network training objectives (mean square error) is set to 10^−2^, (3) the number of training steps is controlled in 6000. The number of hidden layer neurons was determined through repeated verification.

#### 2.3.4. Determination of Peptides ACE-Inhibitory Activity

N-(3-[2-Furyl]Acryloyl)-Phe-Gly-Gly (FAPGG, purchased from Fluka Chemical Corp., Milwaukee, WI, U.S.A.) was used as substrate in ACE-inhibition assay. The reagents were sequentially added in for test reaction according to Table 3 [23]. The absorbance of each reaction solution was determined by a Multiskan Spectrum Microplate Reader at 340 nm. The initial absorbance of blank (*a*1) and sample (*b*1), and the final absorbance (*a*2 and *b*2, after 30 min reaction at 37°C) were recorded. The absorbance decrease of blank and sample are *A* ( = *a*1 − *a*2) and *B* ( = *b*1 − *b*2), respectively. Then, ACE-inhibitory activity (%) was expressed as *I* = (*A* − *B*)/*A*.

#### 2.3.5. Determination of the Degree of Hydrolysis

The degree of hydrolysis (DH) was measured by pH-stat method. The release of amino acids in protein digestion makes pH of the hydrolysate decrease significantly, the alkali solution was added into hydrolysates to maintain pH value. By recording the amount of alkali consumed, the degree hydrolysis of protein and the amount of the rupture protein bonds can be figured out according to the following formula:

(2) $$\text{DH}=\frac{V_{\text{NaOH}}\times N_{\text{NaOH}}}{\alpha \times M_{p}\times h_{\text{hot}}}\times 100\%,$$

where *V*_NaOH_ is consumption volume of alkali (mL) in titration; *N*_NaOH_ is the concentration of alkali (mol/L) in titration; *M*_*p*_ is total protein (g) used; *h*_hot_ is the total number of peptide bonds per gram of protein (mmol/g, for wheat germ protein, taking 7.69); *α* is a-amino acid dissociation degree, it can be calculated according to formula (3):

(3) $$\alpha =\frac{10^{\text{pH}-\text{pK}}}{\left(1+10^{\text{pH}-\text{pK}}\right)},$$

pK is the average pH value of all kinds of amino acids, taking 9.0; pH is response to initial pH.

#### 2.3.6. Analysis of DWGP Amino Acids Composition

Amino acid composition analysis was employed in this study to determine DWGP amino acid composition by o-phthalaldehyde (OPA) precolumn derivatization RP-HPLC determination [24].

## 3. Results and Discussions

### 3.1. Building of QSAR Model on ACE-Inhibitory Peptides

**Figure 2:**
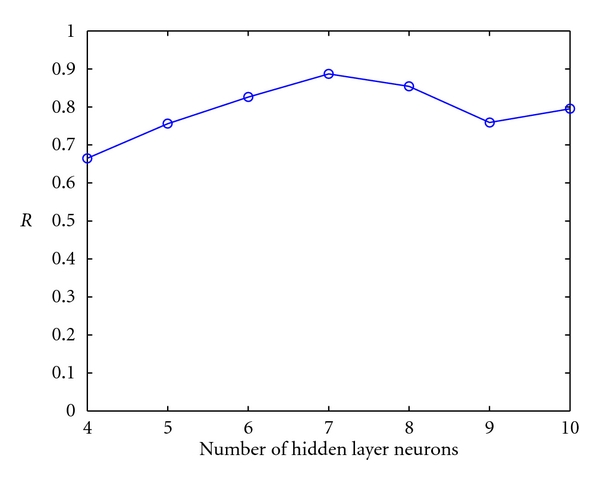
The correlation coefficient *R* values varied with the numbers of hidden layer neurons.

**Figure 3:**
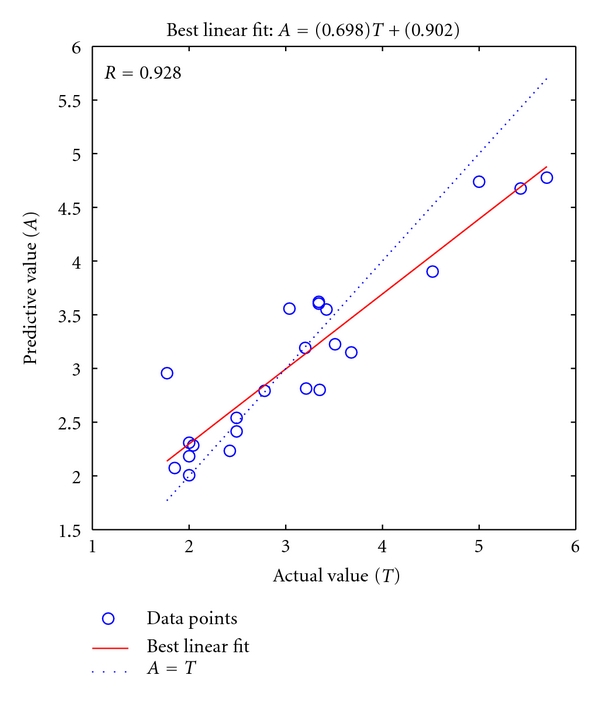
The predicting results of the (6-7-1) BP network model on the set of prediction.

In this study, 4–10 hidden layer neurons were selected to build QASR model, each hidden layer neuron was modeled five times in order to identify the optimal number of hidden layer neurons. Network convergence speed rises when the number of neurons increases, but too many or too few of hidden layer neurons will decrease the generalization performance of model. Under the premise of guaranteed network convergence, a fewer number of neurons are preferred. The correlation coefficients *R* of study samples (the average value of five times of modeling) were shown in Figure 2. It was shown that when the number of hidden layer neurons was 7, the forecast correlation coefficient was the highest. Therefore, seven hidden layer neurons were selected to model the neural network. After repeated modeling, the correlation coefficient *R* reaches to 0.928, the training set mean square error is 0.0188, and the prediction set mean square error is 0.2091. The predicting results of BP network model to the set of prediction were shown in Figure 3.

### 3.2. Structural Features Analysis of ACE-Inhibitory Peptides

The back stepping method was used to find out the operator which has the greatest impact on the activity. The steps are as follows: (1) find out which hidden layer neuron has the greatest impact on output (activity), (2) find out which input neuron (specific *Z* operator) has impact on the found hidden layer neurons. 

**Figure 4:**
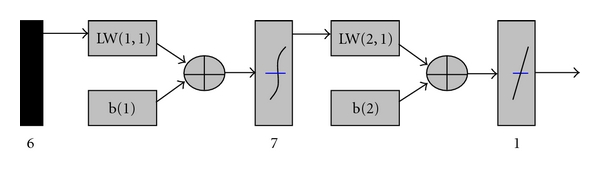
The parametric diagram of BP network model.

In Figure 4, LW(2, 1) refers to the weights when the hidden layer neurons change to the output layer neurons (activity values) through a linear function. If a hidden layer LW(2,1) is bigger, it means that its corresponding neurons in the hidden layer have a greater impact on the output, on the contrary, if LW(2,1) becomes small, its corresponding neurons in the hidden layer have little effect on the output. By searching in the model, the hidden layer neuron with the greatest impact on the output was found. Analysis of the established BP neural network model showed that the weights LW(2,1) value was (0.70466, 0.74384, −0.63652, −0.37093, 0.49303, −1.3532, 1.1885), the sixth hidden layer neurons value (−1.3532) and seventh value (1.1885) have the greatest impact on output. Then, input neurons (*Z* operator) which affected the 6 and 7 hidden layer neurons were investigated.

After searching the hidden layer neurons, the input layer neurons with the greatest impact on the hidden layer neurons were subsequently searched. In Figure 4, LW(1, 1) refers to the weights when the output layer neurons change to the hidden layer neurons. If LW(1,1) becomes bigger, it means the input layer neurons have high impact on the hidden layer neurons; when LW(1,1) becomes smaller, the input layer neurons have little influence on the hidden layer neurons. According to the above searching, we can get the structural features with greater impact on the ACE-inhibitory peptide activity.

Table 4 is weights LW(1,1) of the input layer to hidden layer neuron. Observing the weight values of the various *Z* operators on 6 and 7 hidden layer neurons, we found that the *Z_21_* parameters (*Z_1_* operator of the second amino acid, see numbers in Table 4 with ^†^ superscript) have the greatest impact on the activity, followed by the *Z*_22_ (*Z*_2_ operator on No. 2 position, see numbers in Table 4 with ^‡^ superscript). As we have defined that the *Z*_1_ operator represents the hydrophobicity of amino acids [23], we could draw a conclusion that hydrophobicity of C-terminal amino acids have the greatest influence on ACE-inhibitory activity; and the greater the hydrophobicity is, the higher the ACE-inhibitory activity is. This result is consistent with some previous studies. Wu et al. [11] used *Z* descriptors to investigate quantitative structure-activity relationship of ACE-inhibitory dipeptides, and they found that ACE-inhibitory activity was greatly affected by the three-dimensional chemical properties and hydrophobicity of C-terminal amino acids, that is, the higher the volume and the greater hydrophobicity of amino acids were, the nicer the ACE-inhibitory activity was; so some dipeptides with hydrophobic amino acids at the C-terminal, such as phenylalanine, tryptophan, and tyrosine, will have high ACE-inhibitory activity. Cheung et al. [25] have also shown that if C-terminal was aromatic amino acids and proline, N-terminal was branches aliphatic amino acids; the dipeptides could have high ACE-inhibitory activity. Hellberg et al. measured Cheung' peptides samples in the same laboratory, and modeled the QSAR, he found that the dipeptides with positive charge amino acids at the N-terminal and bulky hydrophobic amino acids at C-terminal would have a stronger ACE-inhibitory activity [26]. As for tripeptides, Wu et al. [11] found that strong hydrophobic and small size of N-terminal amino acids, such as valine, leucine, and isoleucine, were more suitable for high-activity tripeptides; for second amino acid from the N-terminal, small electrical bit, large size, and weak hydrophobicity were more suitable. But for C-terminal, a higher electrical, larger volume, and stronger hydrophobic amino acid was more suitable, such as aromatic amino acids. Through the analysis of the three amino acid ACE-inhibitory peptides, Li [27] also reached a conclusion similar to Wu et al. By analyzing ACE-inhibitory peptides from milk sources, Pripp et al. [7] found that for peptides with less than or equal to 6 amino acids at the C-terminal, the hydrophobicity, the amount of positive charge, and the volume size of amino acids adjacent to the C-terminal greatly affected the ACE-inhibitory peptides activity while the N-terminal amino acid has no direct relationship to the ACE-inhibitory activity. Therefore, the hydrophobicity and size of the C-terminal amino acid have primary effect on ACE-inhibitory activity, and hydrophobic amino acids, aromatic amino acids, or branched-chain amino acids are important components in high-activity peptides. Therefore, protein with high content of hydrophobic amino acid (especially aromatic amino acids) has more potential to produce high activity ACE-inhibitory peptides. By digestion of protein to produce peptides with hydrophobic amino acids at the C-terminal, people will get high ACE-inhibitory activity of hydrolysates.

### 3.3. Amino Acid Composition and Feature Analysis of Wheat Germ Protein Isolates

The DWGP contains 42.84% hydrophobic amino acids (Table 5), it is similar to rice protein isolate, bovine serum albumin, and casein, and is it significantly higher than mung bean protein isolate and peanut protein isolate [27]. Therefore, DWGP is a good protein resource with abundant hydrophobic amino acid. According to the result of quantitative structure-activity relationship analysis that high content of hydrophobic amino acid protein (especially aromatic amino acids) is suitable as protein material to produce ACE-inhibitory peptides (see Section 2.2 of this paper), wheat germ protein isolate is a good material to produce high-activity ACE-inhibitory peptides.

### 3.4. Digestion of Defatted Wheat Germ Protein with Different Proteases

**Figure 5:**
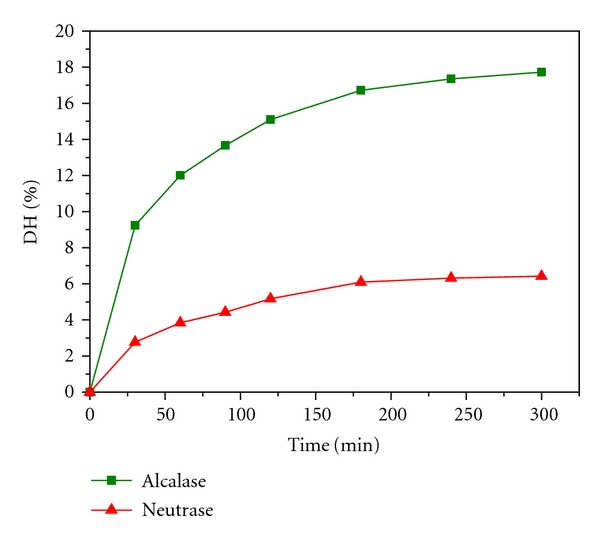
The degree of hydrolysis of DWGP hydrolysate treated with Alcalase and Neutrase.

**Figure 6:**
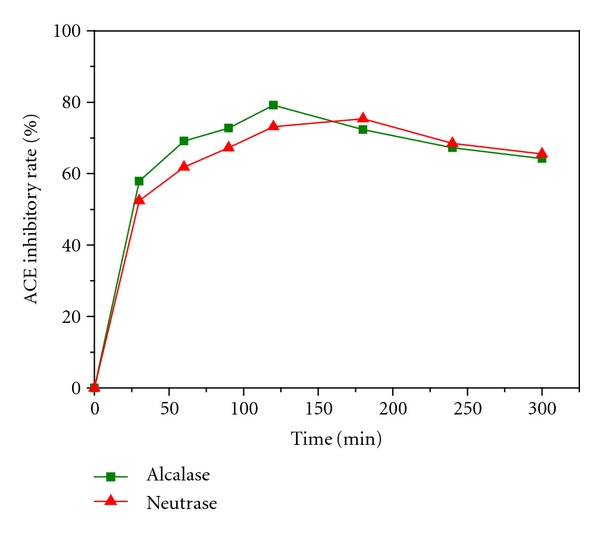
The ACE-inhibitory activity of DWGP hydrolysate treated with Alcalase and Neutrase.

Neutrase (a kind of neutral protease) tends to hydrolyze protein to produce peptides whose C-terminals are hydrophobic amino acids, such as Tyr, Try, or Phe. Alcalase (a kind of alkaline protease) tends to hydrolyze protein to obtain peptides whose C-terminals are amino acids with large side-chain and no charge (aromatic and aliphatic amino acids), such as Ile, Leu, Val, Met, Phe, Tyr, or Trp. Moreover, the hydrolysis process will be accelerated when N-terminals of peptides have hydrophobic amino acids [28, 29]. Proteinase K (EC. 3.4.21.14) acts on Phe, Try, Val, Ile, Leu, Trp, Pro, and Met [30]. Chymotrypsin C (EC 3.4.21.2) acts on Try, Leu, Trp, Pro, Met, Glu, Lys, and Pro [28]. The above proteases all tend to hydrolyze protein to generate peptides with hydrophobic amino acids C-terminals, and the QSAR of ACE-inhibitory peptide studies have shown that peptides which have hydrophobic amino acids C-terminals will show potential strong ACE inhibition, so Neutrase, Alcalase, proteinase K, and chymotrypsin C may be the suitable proteases for high-activity ACE-inhibitory peptides preparation. In addition, Alcalase and Neutrase are microbial enzymes which are easily obtained and low cost compared with proteinase K and chymotrypsin C, so they are suitable for industrial application. In this study, Alcalase and Neutrase were investigated to produce ACE-inhibitory hydrolysates by digest DWGP. The degree of hydrolysis (DH) and the ACE-inhibitory activity of DWGP hydrolysates were presented in Figures 5 and 6, respectively.

From Figure 5, we can find DHs of DWGP digested by either alkaline or neutral protease increased significantly before 120 min, and slightly increased during 120~300 min. Results of Figure 5 imply that the hydrolysis sites of Alcalase and Neutrase are partly similar, but hydrolysis sites of Alcalase exceed Neutrase's; therefore, the former one's hydrolysate has higher DH than the later one's.  Figure 6 shows that the ACE-inhibitory rate of hydrolysates digested by Alcalase is remarkably increased during the preceding 120 min, and then it decreases slowly after 120 min. This result indicated that a long-time digestion might cause the excessive degradation of active peptides. Li observed a similar phenomenon in preparations of ACE-inhibitory peptides from Zein, rice protein isolate, mung bean protein isolate, and peanut protein isolate with Alcalase [27]. Pedroche prepared ACE-inhibitory peptides with Alcalase through hydrolysis of chickpea protein also found that the ACE-inhibitory rate reached the maximum at 30 min and then decreased [31]. From Figure 6, we also find that the inhibitory rate of peptides digested by Neutrase rises during the preceding 180 min, and then decreases slowly. The result also indicated that long-time digestion caused the excessive degradation of active peptides. However, during the preceding 120 min, the ACE-inhibitory activity of the Alcalase hydrolysates was significantly higher than the Neutrase hydrolysates at the same time, and both of them reached almost the same activity level after 120 minutes. According to the average peptide chain length (PLC) formula (PLC = (1/DH) × 100%) of protein digestion [27], higher DH of hydrolysate by Alcalase indicates that more short chain lengths peptides were produced in digestion than by Neutrase. The theoretical conclusion was also proved by the experimental results of Xin et al. [32] and Jia et al. [33], respectively. It has been revealed that the most part of effective ACE-inhibitory peptides after oral administration are small peptides [21], therefore, Alcalase is more suitable for DWGP ACE-inhibitory peptides preparation.

## 4. Conclusions

Based on data of activity, hydrophilic amino acids, three-dimensional shape, size, and electrical parameters of 58 dipeptides, a quantitative structure-activity relationship (QSAR) of amino acids ACE-inhibitory peptides was built with ANN, the related coefficient is 0.928, and by analyzing the ANN model, it was found that (1) C-terminal is primarily important to ACE-inhibitory activity; (2) proteins containing abundant hydrophobic amino acids are potential good source to produce ACE-inhibitory peptides; (3) as for DWGP, Alcalase was a proper protease for ACE-inhibitory peptides preparation.

## Figures and Tables

**Table 1:** *Z* descriptor scores for amino acids.

Amino	Code	*Z_1_*	*Z_2_*	*Z*_3_	Amino	Code	*Z*_1_	Z_2_	*Z*_3_
acid					acid				
Ala	A	0.07	−1.73	0.09	His	H	2.41	1.74	1.11
Val	V	−2.69	−2.53	−1.29	Gly	G	2.23	−5.36	0.30
Leu	L	−4.19	−1.03	−0. 98	Ser	S	1.96	−1.63	0.57
Lie	I	−4.44	−1.68	−1.03	Thr	T	0.92	−2.09	−1.40
Pro	P	−1.22	0.88	2.23	Cys	C	0.71	−0.97	4.13
Phe	F	−4.92	1.30	0.45	Tyr	Y	−1.39	2.32	0.01
Trp	W	−4.75	3.65	0.85	Asn	N	3.22	1.45	0.84
Met	M	−2.49	−0.27	−0.41	GIn	Q	2.18	0.53	−1.14
Lys	K	2.84	1.41	−3.14	Asp	D	3.64	1.13	2.36
Arg	R	2.88	2.52	−3.44	Glu	E	3.08	0.39	−0.07

**Table 2:** The ACE-inhibitory peptides' sequences with *Z* descriptor activity.

Peptide	Log(1/IC_50_)	*Z*_11_*	*Z*_12_	*Z*_13_	*Z*_21_	*Z*_22_	*Z*_23_
AA	3.21	0.07	−1.73	0.09	0.07	−1.73	0.09
AW	5	0.07	−1.73	0.09	−4.75	3.65	0.85
DG	1.85	3.64	1.13	2.36	2.23	−5.36	0.3
GF	3.2	2.23	−5.36	0.3	−4.92	1.3	0.45
GP	3.35	2.23	−5.36	0.3	−1.22	0.88	2.23
GR	2.49	2.23	−5.36	0.3	2.88	2.52	−3.44
GW	4.52	2.23	−5.36	0.3	−4.75	3.65	0.85
GY	3.68	2.23	−5.36	0.3	−1.39	2.32	0.01
IF	3.03	−4.44	−1.68	−1.03	−4.92	1.3	0.45
IW	5.7	−4.44	−1.68	−1.03	−4.75	3.65	0.85
IY	5.43	−4.44	−1.68	−1.03	−1.39	2.32	0.01
RF	3.64	2.88	2.52	−3.44	−4.92	1.3	0.45
RP	1.1818	2.88	2.52	−3.44	−1.22	0.88	2.23
VG	2.96	−2.69	−2.53	−1.29	2.23	−5.36	0.3
VW	1.6	−2.69	−2.53	−1.29	−4.75	3.65	0.85
VY	4.66	−2.69	−2.53	−1.29	−1.39	2.32	0.01
YG	2.7	−1.39	2.32	0.01	2.23	−5.36	0.3
RW	4.8	2.88	2.52	−3.44	−4.75	3.65	0.85
AY	4.28	−2.69	−2.53	−1.29	−4.92	1.3	0.45
RP	3.89	−4.44	−1.68	−1.03	−1.22	0.88	2.23
AF	3.72	0.07	−1.73	0.09	−4.92	1.3	0.45
AP	3.64	0.07	−1.73	0.09	−1.22	0.88	2.23
VP	3.38	−2.69	−2.53	−1.29	−1.22	0.88	2.23
IG	2.92	−4.44	−1.68	−1.03	2.23	−5.36	0.3
GI	2.92	2.23	−5.36	0.3	−4.44	−1.68	−1.03
GM	2.85	2.23	−5.36	0.3	−2.49	−0.27	−0.41
GA	2.7	2.23	−5.36	0.3	0.07	−1.73	0.09
GL	2.6	2.23	−5.36	0.3	−4.19	−1.03	−0.98
AG	2.6	0.07	−1.73	0.09	2.23	−5.36	0.3
GH	2.51	2.23	−5.36	0.3	2.41	1.74	1.11
KG	2.49	2.84	1.41	−3.14	2.23	−5.36	0.3
FG	2.43	−4.92	1.3	0.45	2.23	−5.36	0.3
GS	2.42	2.23	−5.36	0.3	1.96	−1.63	0.57
GV	2.34	2.23	−5.36	0.3	−2.69	−2.53	−1.29
MG	2.32	−2.49	−0.27	−0.41	2.23	−5.36	0.3
GK	2.27	2.23	−5.36	0.3	2.84	1.41	−3.14
GE	2.27	2.23	−5.36	0.3	3.08	0.39	−0.07
GT	2.24	2.23	−5.36	0.3	0.92	−2.09	−1.4
WG	2.23	−4.75	3.65	0.85	2.23	−5.36	0.3
HG	2.2	2.41	1.74	1.11	2.23	−5.36	0.3
GQ	2.15	2.23	−5.36	0.3	2.18	0.53	−1.14
GG	2.14	2.23	−5.36	0.3	2.23	−5.36	0.3
QG	2.13	2.18	0.53	−1.14	2.23	−5.36	0.3
SG	2.07	1.96	−1.63	0.57	2.23	−5.36	0.3
LG	2.06	−4.19	−1.03	−0.98	2.23	−5.36	0.3
GD	2.04	2.23	−5.36	0.3	3.64	1.13	2.36
TG	2	0.92	−2.09	−1.4	2.23	−5.36	0.3
EG	2	3.08	0.39	−0.07	2.23	−5.36	0.3
PG	1.77	−1.22	0.88	2.23	2.23	−5.36	0.3
LA	3.51	−4.19	−1.03	−0.98	0.07	−1.73	0.09
KA	3.42	2.84	1.41	−3.14	0.07	−1.73	0.09
RA	3.34	2.88	2.52	−3.44	0.07	−1.73	0.09
YA	3.34	−1.39	2.32	0.01	0.07	−1.73	0.09
FR	3.04	−4.92	1.3	0.45	2.88	2.52	−3.44
HL	2.49	2.41	1.74	1.11	−4.19	−1.03	−0.98
DA	2.42	3.64	1.13	2.36	0.07	−1.73	0.09
EA	2	3.08	0.39	−0.07	0.07	−1.73	0.09
DM	2.7782	3.64	1.13	2.36	−2.49	−0.27	−0.41
IP	3.89	2.92	−4.44	−1.68	−1.22	0.88	2.23

*Z_*mn*_ the first number (*m*) behind *Z* represents the sequence of the amino acid in peptide, and the second number (*n*, from 1 to 3) represents the hydrophilic amino acids, three-dimensional shape, size, and electrical parameters, respectively.

**Table 3:** Reagents used in determination of ACE inhibiting activity.

	Blank (*μ*L)	Sample (*μ*L)
ACE (0.1 U/mL)	10	10
FAPGG (1 mmol/L)^1^	50	50
HEPES buffer^2^	40	0
Sample	0	40

^1^FAPGG (1.0 mmol/L): prepared with 0.08 M HEPES buffer (pH 8.3) containing 0.3 M NaCl.

^2^HEPES buffer: HEPES 1.910 g, NaCl 1.755 g, dissolved with double-distilled water, pH adjusted with NaOH, and metered volume with double-distilled water to 100 mL, stored at 4°C.

**Table 4:** The neurons weights from input layer to hidden layer.

Neurons	*Z*_11_*	*Z*_12_	*Z*_13_	*Z*_21_	*Z*_22_	*Z*_23_
(1)	−0.3515	0.28331	0.28286	−0.44043	0.037431	−0.085447
(2)	0.27249	0.041643	−0.9822	−0.087255	0.47412	−0.18318
(3)	0.57416	−0.14092	0.60775	−0.08924	0.11108	−0.5922
(4)	0.24392	0.21597	−0.20115	0.24221	−0.07429	−0.54316
(5)	0.23817	0.07212	0.68217	−0.00285	0.26846	−0.47522
(6)	−0.30588	0.21639	0.036361	−0.41676^†^	−0.2514^‡^	−0.23479
(7)	−0.090315	−0.058403	0.0745	−0.32458^†^	−0.24483^‡^	−0.10795

*Z_*mn*_ the first number (*m*) behind *Z* represents the sequence of the amino acid in peptide, and the second number (from 1 to 3) represents the hydrophilicity of amino acids, three-dimensional shape, size, and electrical parameters, respectively.

**Table 5:** Amino acid composition of wheat germ protein isolates (g/100 g protein).

Amino acid	Content
Asp +Asn	8.40
Glu + Gln	15.28
Ser	4.40
His	3.15
Gly	6.19
Thr	3.94
Arg	9.79
Ala	6.41
Tyr	2.97
Cys	0.39
Val	7.20
Met	1.70
Phe	5.22
Ile	4.94
Leu	8.07
Lys	6.33
Pro	5.63
Trp	0.69
Hydrophobic amino acids	42.84
Aromatic amino acids	8.89
